# Your Synapse or Mine? Promiscuous Protein Exchanges between Neighboring Synapses

**DOI:** 10.1371/journal.pbio.0040297

**Published:** 2006-08-15

**Authors:** Françoise Chanut

Synapses, the connections that link neurons into circuits, can be plastic or stable in the mammalian brain. Right after birth, synapses form and dissolve among nascent neurons at breakneck speed as the animal adapts to its new surroundings. But, over time, while some plasticity remains and allows for learning, most synapses stabilize and some may last a lifetime. How synapses are maintained over such long periods is somewhat of a mystery, especially in light of the fact that structural proteins constantly move in and out of synapses. In theory, the active turnover of synaptic components might simply reflect the balance between protein synthesis and degradation. But, in a recent study, Shlomo Tsuriel, Ran Geva, Noam Ziv, and their colleagues find that two prominent synaptic proteins, Synapsin I and ProSAP2, turn over primarily through rapid exchanges between neighboring synapses, rather than via synthesis and degradation. These observations add an interesting twist to the already complex picture of synapse biology.

Synapses are specialized devices that serve to transfer electrical impulses between neurons. They form at discrete contact points between the neuron’s main branch (the axon) and the complex arborizations (dendrites) that sprout from its target neuron’s cell body. A number of specialized structures and molecules accumulate at synapses, including synaptic vesicles chock-full of neurotransmitters on the axonal (presynaptic) side, and neurotransmitter receptors on the dendritic (postsynaptic) side. Synapsin I and ProSAP2 play important structural roles: Synapsin I tethers synaptic vesicles underneath the presynaptic lipid membrane and ProSAP2 organizes the postsynaptic architecture.

To follow the whereabouts of Synapsin I and ProSAP2, the researchers tagged each protein with fluorescent dyes and coaxed cultured neurons from the hippocampus (a brain region involved in learning) of newborn rats to synthesize these fluorescently tagged proteins. As the neurons grew in culture, they established synapses that incorporated the tagged Synapsin I or ProSAP2. The synapses were easily visualized as bright fluorescent spots studding dendrites and axon branches. The first dye, called green fluorescent protein (GFP, a small protein that was originally isolated from jellyfish), fluoresces readily but can be extinguished with intense illumination, a phenomenon called photobleaching. The researchers photobleached individual synapses containing GFP-tagged Synapsin I or ProSAP2 with an intense laser beam. Over time, a fluorescent signal reappeared at the bleached synapses, indicating that bleached proteins were replaced with tagged proteins from unbleached areas. Tagged Synapsin replenished bleached synapses in about 40 minutes, and tagged ProSAP2 in two to four hours.

But these experiments did not show where the replenishing proteins came from. To answer this question, the researchers took advantage of a second dye, photoactivatable variant of GFP (PA-GFP), whose fluorescence is activated, rather than extinguished, with intense illumination. The researchers photoactivated PA-GFP-tagged Synapsin I or ProSAP2 over small portions of dendrites or axons. Over the course of 10 to 40 minutes, fluorescence gradually declined at the illuminated synapses, and concomitantly increased in neighboring synapses. These results indicate that pre- and postsynaptic proteins routinely hop from one synapse to the next with timescales of tens of minutes, a behavior that might account for the rapid replenishment of photobleached synapses.

Still, some of the replenishing material could also have come from new protein synthesis. By tracking PA-GFP-tagged proteins from cell bodies, where most synthesis typically occurs, into dendrites and axons, the researchers determined that newly synthesized Synapsin I and ProSAP2 moved too slowly to explain the rapid replenishment of bleached synapses. In addition, inhibitors of protein synthesis and degradation did not significantly affect the synapses’ replenishment rates, confirming that the high turnover rate of Synapsin I and ProSAP2 owes mostly to local exchanges among neighboring synapses.

How the promiscuous exchange of structural proteins such as Synapsin I and ProSAP2 affects synaptic stability is still unclear. Competition for a local pool of synaptic components could eventually determine which synapse is stabilized. Curiously, synaptic signaling may be a destabilizing factor in the young hippocampal neurons, as electric stimulations to the cultures greatly increased Synapsin I and ProSAP2 trafficking. Whether local promiscuity is a characteristic of youthful synapses or also holds true for more mature ones remains to be seen.

## 

**Figure:**
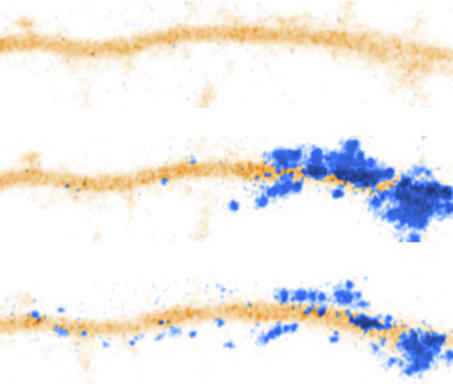
Photoactivation of synapses on one side of a dendritic segment (orange) is followed by migration and incorporation of photoactivated PA-GFP-tagged ProSAP2 (blue) into neighboring synapses

